# Cell-Type Specific Inhibition Controls the High-Frequency Oscillations in the Medial Entorhinal Cortex

**DOI:** 10.3390/ijms232214087

**Published:** 2022-11-15

**Authors:** Shalva Gurgenidze, Peter Bäuerle, Dietmar Schmitz, Imre Vida, Tengis Gloveli, Tamar Dugladze

**Affiliations:** 1 Cellular and Network Physiology Group, Neuroscience Research Center, Charité-Universitätsmedizin Berlin, Corporate Member of Freie Universität Berlin, Humboldt-Universität Berlin, and Berlin Institute of Health, Charitéplatz 1, 10117 Berlin, Germany; 2 Institute of Integrative Neuroanatomy, Charité-Universitätsmedizin Berlin, Corporate Member of Freie Universität Berlin, Humboldt-Universität Berlin, and Berlin Institute of Health, Charitéplatz 1, 10117 Berlin, Germany; 3 Neuroscience Research Center, Charité-Universitätsmedizin Berlin, Corporate Member of Freie Universität Berlin, Humboldt-Universität Berlin, and Berlin Institute of Health, Charitéplatz 1, 10117 Berlin, Germany; 4 German Center for Neurodegenerative Diseases (DZNE) Berlin, Charitéplatz 1, 10117 Berlin, Germany; 5 Max Delbrück Center for Molecular Medicine in the Helmholtz Association, Robert-Rössle-Straße 10, 13125 Berlin, Germany

**Keywords:** inhibitory synaptic transmission, opioid signaling, cortical microcircuits, oscillatory network activity, interneurons, pyramidal cells

## Abstract

The medial entorhinal cortex (mEC) plays a critical role for spatial navigation and memory. While many studies have investigated the principal neurons within the entorhinal cortex, much less is known about the inhibitory circuitries within this structure. Here, we describe for the first time in the mEC a subset of parvalbumin-positive (PV+) interneurons (INs)—stuttering cells (STUT)—with morphological, intrinsic electrophysiological, and synaptic properties distinct from fast-spiking PV+ INs. In contrast to the fast-spiking PV+ INs, the axon of the STUT INs also terminated in layer 3 and showed subthreshold membrane oscillations at gamma frequencies. Whereas the synaptic output of the STUT INs was only weakly reduced by a μ-opioid agonist, their inhibitory inputs were strongly suppressed. Given these properties, STUT are ideally suited to entrain gamma activity in the pyramidal cell population of the mEC. We propose that activation of the μ-opioid receptors decreases the GABA release from the PV+ INs onto the STUT, resulting in disinhibition of the STUT cell population and the consequent increase in network gamma power. We therefore suggest that the opioid system plays a critical role, mediated by STUT INs, in the neural signaling and oscillatory network activity within the mEC.

## 1. Introduction

The morphological and electrophysiological characterization of the superficial layer cells in the medial entorhinal cortex (mEC), as an information transfer station to and from the hippocampus, was initiated more than two decades ago [1,2,3,4,5,6,7]. The interest in the structures of the entorhinal cortex (EC) increased dramatically due to later studies demonstrating its critical role in spatial navigation and memory. The principal cells of the mEC superficial layers generate internal spatial representation, e.g., when the neurons functionally act as grid or border cells [8,9,10]. Furthermore, the formation of episodic memory crucially depends on the hippocampal formation and its afferents from the EC [11]. The EC input to the hippocampus is organized via two major pathways from the superficial mEC, the perforant path to the dentate gyrus (DG) originating from EC layer 2 (L2), as part of the trisynaptic pathway that continues through the cornu ammonis (CA)3-CA1 and back to the EC, and the monosynaptic temporoamonic projection to the CA1/Subiculum from EC layer 3 (L3). The mEC superficial layers comprise two principal cell populations: the reelin-positive stellate (SC) and the reelin-negative pyramidal (PC) cells [1,7]. While the SCs are mainly confined to L2, the PCs are located in both L2 and L3. It has been shown that L3 PCs, which provide the main input to the CA1/Subiculum [12], are crucial for temporal association memory [13] and are relevant for gamma frequency network oscillations [14].

GABAergic interneurons (INs) provide important inhibition to the neural circuits of the EC and thus contribute to spatial navigation and memory performance. Cortical INs can be largely classified into three main non-overlapping groups defined by one of the three different biomarkers, parvalbumin (PV), somatostatin (SOM), or the inotropic serotonin receptor (5HTR3a) [15,16]. This fundamental classification can also be applied to the EC [17,18,19,20,21,22,23,24]. From a functional point of view, PV-positive INs (PV+ INs) represent the most important and the best investigated subset of the GABAergic cells within the mEC because they provide recurrent inhibition, e.g., directly inhibiting firing in the grid cells, border cells, and head direction cells [17], suggesting a critical role in spatial representation. However, in contrast to the principal cells of the superficial mEC, whose intrinsic properties have been extensively investigated [1,2,3,4,5,25,26,27,28,29], information about the intrinsic and the firing properties of the distinct IN classes and their functional meaning is sparse [2,18]. This is especially true for the role of certain IN types in oscillatory network activities, which are central to EC function.

Network oscillation in the gamma frequency range is thought to coordinate the spike timing of the neuronal ensembles across brain regions, such as, e.g., between the EC and the hippocampus [30]. The PV+ INs that provide the majority of the local inhibition within the mEC superficial layers [31] are essential for such oscillations. These INs express μ-opioid receptors in the hippocampus [32,33] and cortex [34], and the activation of these receptors may control the excitability of the feed-forward INs [32,35]. In the hippocampus, μ-opioid receptor activation suppresses the feed-forward inhibition-driven gamma rhythm, leading to a loss of phase coupling between the CA3 and CA1 gamma oscillations [36]. A decrease in oscillatory synchronization in the EC and the disruption of functional connectivity between CA1 and the mEC was also observed by activation of the cannabinoid type-1 receptors that are expressed on cholecystokinin-positive INs with a regular firing behavior [37]. In addition, the disruption of sensory gating and neural oscillations in the theta band was reported by the activation of these receptors [38]. Thus, both modulatory systems, the opioid and the cannabinoid receptors, impact network oscillatory behavior, albeit based on different mechanisms and with distinct cell targets. While cannabinoid type-1 receptor-expressing INs selectively innervate the principal cells of L2 that project exclusively outside the hippocampus [39], the μ-opioid-expressing PV+ INs innervate both the mEC principal cell populations in L2, the SCs and the PCs, that project to the hippocampus and outside the hippocampus, respectively [40]. Hence, the μ-opioid receptors expressed in the mEC may influence oscillatory network behavior and affect EC–hippocampal communication, with a potential impact on learning and memory. Despite the presumed importance for the mEC, the cellular mechanisms underlying the modulation of the EC neuronal network activity by these receptors remain unknown. In particular, this is true for the L3 PCs, the main EC input to the CA1/Subiculum [12].

In the present study, we addressed the modulatory functions of the μ-opioid system on the GABAergic system. We investigated the effects of the pharmacological activation of μ-opioid receptors on the inhibitory synaptic transmission onto the L3 PCs and the GABAergic INs, as well as their impact on the oscillatory network activity within the superficial mEC. Our results show that L2-3 INs are diverse, and the opioid system acts on the inhibitory synaptic transmission in a cell-specific manner.

## 2. Results

### 2.1. Anatomical and Electrophysiological Features of INs in the mEC

**Figure 1:**
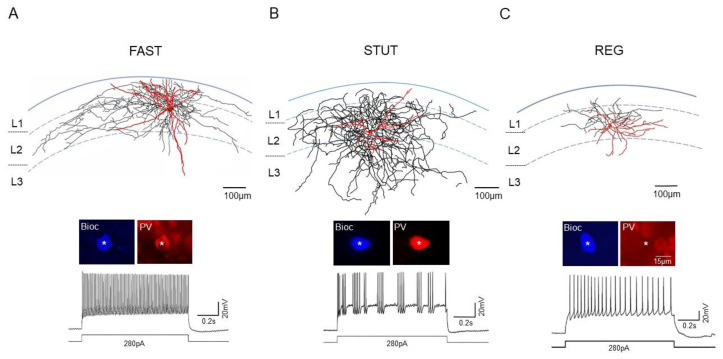
The properties of morphologically and immunocytochemically identified interneurons. (**A**–**C**, **upper**) Neurolucida reconstruction of the biocytin-filled representative PV-immunopositive FAST (**A**) and STUT (**B**) as well as PV-immunonegative REG (**C**). Dendrites are shown in red and axons in black. These INs were selected in GFP mice based on the fluorescence signals before they underwent final identification via immunostaining for PV (**A**–**C**, **insert**, somas are marked with asterisk). (**A**–**C**, **bottom**) Examples of FAST, STUT, and REG voltage responses to depolarizing current steps of 280 pA. Note a typical fast (**A**), stuttering-like (**B**), and regular (**C**) firing of FAST, STUT, and REG, respectively.

In cortical circuits, inhibition to the principal cells is provided by diverse types of GABAergic INs. To characterize the distinct types in the superficial layers (L1-3) of the mEC, we performed whole-cell patch-clamp recordings in transverse EC–hippocampal combined slices from mice, followed by the immunocytochemical and morphological characterization of the recorded and intracellularly filled cells (Figure 1). The INs were selected for the recordings either in PV-GFP mice, based on their fluorescence signals, or in wild-type mice, based on the multipolar appearance of their cell bodies. In our sample, we found three distinct groups of INs with respect to their PV-immunoreactivity and firing behavior upon the depolarizing current injection (DCI): (1) fast-spiking PV-positive INs with high-frequency non-adapting discharge pattern (‘FAST’): (2) stuttering PV-positive (‘STUT’) INs with high-frequency irregular bursting [41]; and (3) regular-spiking PV-negative INs with slower and adapting firing (‘REG’) (Figure 1). 

**Figure 2:**
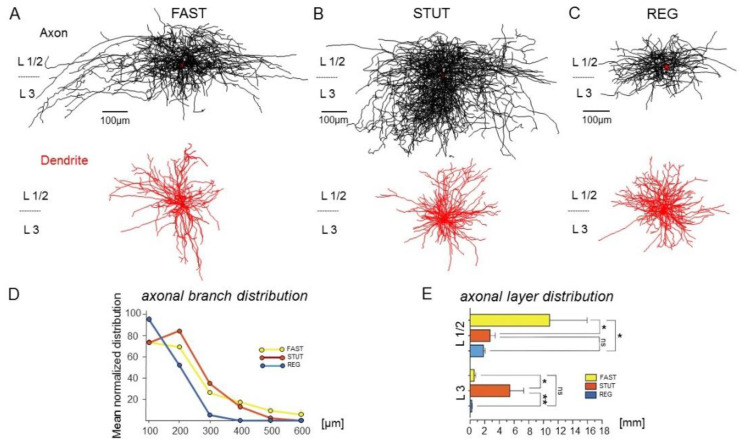
Morphological features of FAST, STUT, and REG. (**A**–**C**) Superimposed Neurolucida reconstruction of 9 cells each for FAST (**A**), STUT (**B**), and REG (**C**). While the dendrite morphology (in red) in all IN types was broadly similar, the axons (in black) of FAST and REG formed typical arbors, restricted mainly within the L1-2, whereas the STUT cells axon also has a prominent axonal arborization in L3. Therefore, STUT cells exhibited extensively arborized, long-ranging axons distributed at a larger distance in L2-3, both horizontally and vertically. Soma is depicted within the axonal reconstruction in red. (**D**) Mean normalized distribution of axonal branches evaluated by Sholl analysis using six concentric nested spherical shells (each 100 μm) in successive radial distance from the soma. (**E**) Summary bar charts showing the axonal layer-specific distribution of FAST (yellow bars), STUT (orange bars), and REG (blue bars). *p* values are given as ns (*p* > 0.05), * (*p* ≤ 0.05) and ** (*p* ≤ 0.01).

Inspection of the biocytin-filled and visualized INs suggested differences in their morphological features. Therefore, we analyzed the distribution of their axons and dendrites (Figure 2). The PV+ FAST had radially oriented dendrites with a total length of 4.12 ± 2.1 mm (8 INs), spanning the superficial layers but often extending into deeper layers of the mEC (Figure 1A and Figure 2A). The axon formed a typical horizontal arbor in L1 and L2 (L1/2), with a total length of 11.4 ± 5.32 mm. Axon collaterals were found around the cell bodies of these layers (Figure 2A), which was consistent with these INs being basket cells [22].

The PV+ STUT had a smaller but more spherical dendritic distribution (total length 1.78 ± 0.43 mm, n = 9) confined to the upper layers (Figure 2B). While their axon had a total length of 8.1 ± 2.28 mm, similar to the FAST (*p* = 0.27), and showed a wide horizontal spread, it had a broader vertical distribution, covering all three superficial layers of the mEC (Figure 2B). As with the FAST, axon collaterals were found around the cell bodies in these layers. 

The REG had a compact somato-dendritic domain with a total dendrite length of 2.19 ± 0.26 mm (n = 9), which was comparable to the FAST and STUT (*p* = 0.17 and *p* = 0.22, respectively, Figure 2C). Their axon, however, with a length of 2.04 ± 0.24 mm, was substantially shorter than those of the FAST and STUT (*p* = 0.04 and *p* = 0.011, respectively) and had limited horizontal and vertical spread, mainly localized in L1/2 (Figure 2C).

As the three interneuron types diverged in their axonal distribution, we extended the morphological analysis by examining the branching pattern and laminar distribution of the axon. Sholl analysis (Figure 2D) showed that the axonal branches of the REG were concentrated within 100 μm around the soma and dropped off very quickly beyond this distance. In contrast, the FAST and STUT had more extensively branching, longer-range axons that showed a dense axonal arborization within 200 μm around the soma, but collaterals could extend over 400 μm. Moreover, substantial differences between these IN types were found in the vertical, layer-specific distribution of the axons (Figure 2E). Within L1/2, the predominant axonal arborization was featured by the FAST (10.84 ± 5.11 mm vs. 2.71 ± 0.72 mm and 1.81 ± 0.23 mm for the STUT and REG, *p* = 0.048 and *p* = 0.040, respectively), while no significant differences were found between the STUT and REG (*p* = 0.136). Thus, FAST INs may potentially be involved in EC–hippocampal interaction via the trisynaptic pathway originating from L2. In marked contrast, within layer 3, STUT exhibited significantly stronger axonal arborization than the two other IN types (5.42 ± 1.90 mm vs. 0.55 ± 0.23 mm and 0.23 ± 0.06 mm for FAST and REG, *p* = 0.018 and *p* = 0.009, respectively, Figure 2E), with no significant differences between the FAST and REG (*p* = 0.086). The predominant vertical axonal spread of the PV-positive STUT compared to the FAST and REG INs suggests that this type is the main source of perisomatic inhibition for the L3 PCs.

### 2.2. Intrinsic Physiological Properties of mEC INs

We first compared the passive and active membrane properties of the two morphologically distinct PV+ INs to clarify whether they also show differences in their physiological properties. The two types had a similar mean resting membrane potential (FAST: −60.44 ± 1.48 mV vs. STUT: −63.73 ± 1.93 mV, *p* = 0.20), membrane input resistance (179.3 ± 14.2 MΩ vs. 230.5 ± 22.5 MΩ, *p* = 0.08), and AP threshold (−41.1 ± 1.2 mV vs. −44.98 ± 1.59 mV, *p* = 0.08). However, the FAST interneurons had a significantly faster membrane time constant (8.0 ± 0.83 ms vs. 13.1 ± 2.0 ms, *p* = 0.04), shorter AP duration (half-width: 0.57 ± 0.06 ms vs. 0.77 ± 0.02 ms, *p* = 0.01), and smaller SAG (−1.78 ± 0.56 mV vs. −4.10 ± 0.89 mV, *p* = 0.04, n = 9 and n = 11, respectively). The most distinctive electrophysiological differences between these INs was the subthreshold membrane potential oscillations observed in the STUT in addition to the defining “stuttering” firing pattern independent of the amplitude of the DCI. In contrast, the FAST showed no subthreshold oscillations and a higher frequency tonic firing in response to the suprathreshold DCI (see below). Taken together, the data indicate that FAST and STUT comprise two distinct subsets of PV+ INs in the superficial mEC, as already suggested for other brain areas [42]. The putative distinct postsynaptic target of the two PV+ INs reflected by the divergence in their axonal arborization pattern suggests that STUT represents a separate, previously undescribed subtype of PV+ INs in the mEC.

As PV-positive FAST are the best characterized INs in several brain areas, including the mEC [22,31], we focused on the properties of STUT, as a previously undescribed PV-positive interneuron subtype in the mEC together with the also poorly described PV-negative REG INs in this area to fill these gaps.

**Figure 3:**
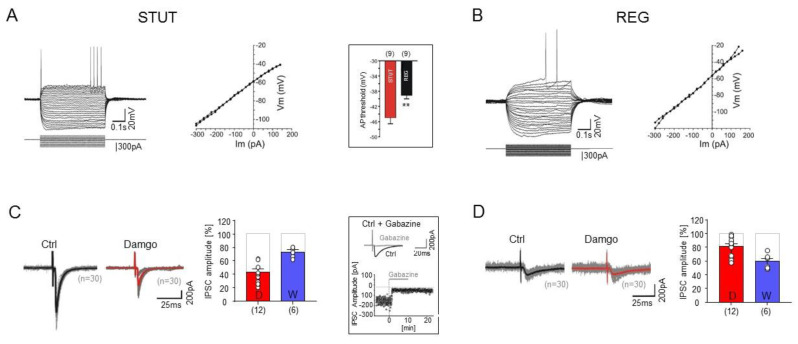
Effects of μ-opioid and cannabinoid receptor activation on IPSCs in GABAergic interneurons of superficial mEC. (**A**,**B**) Examples of STUT (**A**) and REG (**B**) voltage responses to hyperpolarizing and depolarizing current steps of different amplitudes (from −300 pA to 280 pA, 20 pA steps, 500 ms, **left**) with corresponding I-V plots (**right**). Inset (framed) depicts a clear difference in AP threshold between these two IN types. *p* value is given as ** (*p* ≤ 0.01). (**C**,**D**) Example of opioid agonist damgo on IPSCs of INs [30 individual traces (n = 30) for IPSCs are shown in gray, and superimposed averaged trace is shown in black (control) and in red (damgo)], as well as summary bar charts of the reduced peak amplitudes for opioid (damgo, in red) and cannabinoid (WIN, in blue) agonists. Note faster inhibitory synaptic events in STUT than those in REG. Inset (framed) shows that IPSCs were completely abolished in the presence of the GABA_A_ receptor antagonist gabazine.

In terms of intrinsic physiological properties (Figure 3), the PV-negative REG showed resting membrane potentials (−61.30 ± 1.71 mV, *p* = 0.40), membrane input resistance (273.1 ± 25.29 MΩ, *p* = 0.23), and sag potentials upon −300 pA negative current injections (−5.43 ± 1.56 mV, *p* = 0.45) that were similar to those of the PV-positive STUT (see above), which is also reflected in the comparable voltage–current relationships (Figure 3A,B). However, the two types differed in other features: REG had a significantly longer membrane time constant (27.85 ± 4.98 ms, *p* = 0.007) and AP duration (half-width: 1.04 ± 0.08 ms, *p* = 0.002, n = 8). Examination of the AP threshold of these two interneuron groups upon DCI revealed significantly higher values for REG in comparison to STUT (−39.13 ± 0.89 mV, *p* = 0.007, Figure 3). Finally, as a key difference in their firing pattern, STUT demonstrate “stuttering”-like firing, in marked contrast to the regular firing of REG with substantial spike frequency adaptation. The irregular bursting firing, accompanied by subthreshold membrane potential oscillations (see below), was the exclusive hallmark of STUT but not the other IN groups (see also [41]).

### 2.3. Divergent Effects of Opioid and Cannabinoid Receptor Agonists on STUT and REG

μ-opioid receptors are abundantly expressed in the mEC [34,43] and modulate inhibitory transmission onto principal cells [31]. To examine the properties of synaptic transmission and its modulation by these receptors, we recorded isolated GABAergic monosynaptic IPSCs in a voltage clamp mode at –70 mV, evoked by extracellular stimulation in the close vicinity of the recorded cells (Figure 3). Ionotropic glutamatergic transmission was antagonized by bath-applied ionotropic glutamate receptor antagonists (CNQX, 10 μM; APV, 60 μM). An examination of the IPSCs on the two IN types revealed that the monosynaptic IPSCs in these cell types were clearly different, with an average half-width of 3.83 ± 0.35 ms vs. 8.68 ± 0.39 ms in the STUT and REG, respectively (*p* < 0.0001). The effects of the opioid receptor agonist were examined in the INs of both groups (Figure 3C,D). In the presence of bath-applied damgo (200 nM), the IPSC amplitude in the STUT was markedly more strongly attenuated (to 42.94 ± 3.94% of control, n = 12) than in the REG (to 80.75 ± 4.21%, n = 12, *p* = 0.0001, Figure 3C,D).

In an additional set of experiments, we examined the effect of the cannabinoid receptor agonist WIN 55.212 (WIN, 1 μM) on the monosynaptic IPSCs in the two IN types. In contrast to damgo, WIN affected the inhibitory synaptic responses in the STUT to a lesser degree (reduced to 72.92 ± 3.11% of control, n = 6) than in the REG (to 59.17 ± 4.4% of control, n = 6, *p* = 0.029, Figure 3C,D). The effects of damgo and WIN were antagonized by the opioid and cannabinoid receptor antagonists STAP (500 nM) and AM 251 (5 μM), respectively, in all tested cells (n = 12). Furthermore, we confirmed that the extracellular stimulation-evoked monosynaptic IPSCs were mediated by GABA_A_ receptors, because they were completely abolished by the GABA_A_ receptor antagonist gabazine (SR-95531, 10 μM, 4 INs of both type, Figure 3).

### 2.4. Effects of μ-Opioid and Cannabinoid Receptor Agonists on the Inhibitory Synaptic Transmission onto PCs

**Figure 4:**
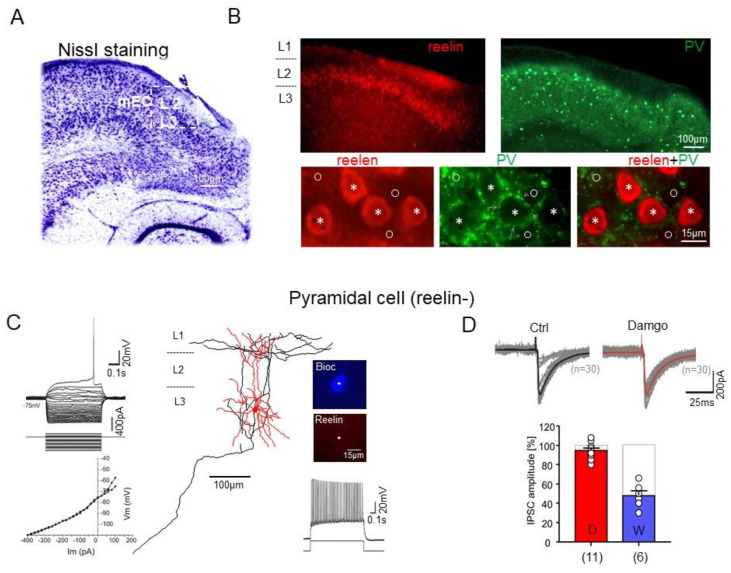
Properties of the mEC L3 pyramidal cells. (**A**). Nissl staining of the EC. (**B** (**upper**)) Reelin-immunopositive stellate cells (**left**, ‘reelin’) and PV-immunopositive interneurons (**right**, ‘PV’) in superficial mEC. (**B** (**bottom**)) PV-immunopositive axon terminals (**middle**, ‘PV’, arrows) surround the somata of both reelin-immunopositive (asterisks) and reelin-immunonegative (circle) principal cells in superficial mEC. (**C**) Morphology of a biocytin-filled L3 reelin-negative (reelin-, **middle**) PC. Examples of the voltage responses (**C**, **left**, **upper**) upon hyperpolarizing and depolarizing current injection and corresponding current–voltage (I–V) relations (**left**, **bottom**) for each voltage response in **C**. Note, no sag potential and later rheobase firing of PC (**C**, **left**). Firing of this cell upon depolarizing 280 pA current injection (**right**, **bottom**). The PC was immunonegative for reelin (reelin−, **C**, **right**, **upper**). (**D**) Effects of μ-opioid receptor agonist damgo on the inhibitory transmission in reelin-negative PC (**upper**). The stimulation-evoked IPSCs were slightly, but not significantly, affected in these cells. The corresponding summary bar charts of the peak amplitudes of IPSCs obtained after application of the opioid (in red) and cannabinoid (in blue) receptor agonists (**bottom**).

The superficial mEC contains two distinct principal cell populations: SCs and PCs, which can be distinguished by the presence or absence of reelin expression, respectively. Therefore, we applied immunostaining for reelin to separate these cell populations (Figure 4). We found that the reelin-positive cell bodies were mostly located in L2, whereas the reelin-negative cells were present in both layers 2 and 3, with a preference for L3, which is consistent with the laminar distribution of the two principal cell types. Double immunostaining for reelin and PV further showed that the axon collaterals were localized around the reelin-positive as well as the reelin-negative somata (Figure 4B), indicating that both SCs and PCs are innervated by PV+ INs, as previously reported for L2 [39].

In an earlier study, we demonstrated a strong suppression (up to 80%) of IPSCs mediated by FAST PV-positive BCs in L2 SCs by the μ-opioid receptor agonist damgo [31]. In the present study, we examine the modulation of IPSCs by these receptors in L3 PCs, which are the preferential targets of STUT (see above). Consistently with previous descriptions [2,3,4,5], reelin-negative PCs had an apical dendrite with a tuft within the superficial layers and multiple basal dendrites, as well as a long-range axon projecting through the angular bundle towards the dentate gyrus (Figure 4C). The monosynaptic IPSCs evoked by extracellular stimulation in these cells had a mean amplitude of −234.7 ± 29.48 pA (n = 11). When the μ-opioid receptor antagonist damgo (200 nM) was bath applied, the IPSC amplitude showed a very small reduction in PCs (to 94.64 ± 2.45% of control, n = 11, *p* = 0.06). To control for the presynaptic modulation of inhibitory transmission, we examined the effects of the bath-applied cannabinoid receptor agonist WIN (1 μM) on the monosynaptic IPSCs in the PCs and observed a strong attenuation of the mean peak amplitude (to 47.83 ± 5.19% of control, n = 6, *p* = 0.0001) (see also [39]) (Figure 4D). Hence, while cannabinoid receptors directly and strongly influence IN-PC inhibitory synaptic transmission, μ-opioid receptor activation does not have a strong impact on the monosynaptic IPSCs in L3 PCs. The strong suppression of IPSCs in the presence of the μ-opioid agonist in STUT (see above) but not in the PCs indicates a strong attenuation in the IN–IN interaction through μ-opioids, with no substantial effects on the direct STUT IN-L3 PC interaction.

### 2.5. Intrinsic Membrane Properties of STUT Support Their Involvement in Oscillatory Network Activity within the mEC

**Figure 5:**
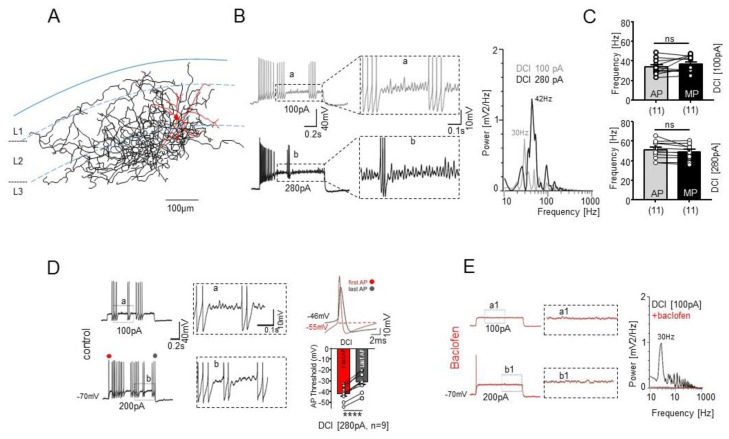
Subthreshold membrane potential oscillations and firing of STUT. (**A**) Neurolucida reconstruction of a biocytin-filled PV-immunopositive STUT. (**B**) Subthreshold oscillations in STUT upon depolarizing current injection with different intensity (**left**) and the corresponding power spectra (**right**). The peak frequency of membrane oscillations varied depending on the intensity of injected pulses but remained at the gamma frequency range (30 Hz and 42 Hz). a, b (framed, **middle**)—enlarged display of a, b (framed, **left**). (**C**) Summary bar charts of the peak frequency of subthreshold membrane oscillations and firing of APs upon 100 pA and 280 pA DCI. Note no significant population difference between subthreshold oscillations and AP firing frequency. *p* value is given as ns (*p* > 0.05). (**D**) Examples of STUT responses upon 100 pA and 200 pA DCI. Upon DCI, STUT generate one AP or burst of spikes interrupted by subthreshold gamma band membrane potential oscillations followed by AP trains at the same gamma frequency range (**left**). a, b (framed, **middle**)—enlarged display of a, b (**left**). The first (●) and last (●) APs possess significantly different AP threshold (**right**). *p* value is given as **** (*p* ≤ 0.0001). (**E**) Effect of GABA_B_ receptor agonist baclofen on the intrinsic membrane properties of STUT held at the same membrane potential (−70 mV) as those in control in (**D**). Baclofen strongly suppressed the intrinsic oscillations and AP firing (**left**). a1, b1 (framed, **middle**)—enlarged display of a1, b1 (framed, **left**). The corresponding power spectra of subthreshold oscillations upon 100 pA DCI before (black) and after (red) baclofen application.

We next examined whether the intrinsic properties of STUT may support the network activities. In this respect, one unique characteristic feature of STUT was their ability to generate subthreshold fluctuation of the membrane potentials upon DCI (Figure 5B–D), which distinguishes them from REG and all the other IN classes in the superficial mEC studied so far [18,19,20]. The frequency of the subthreshold oscillations in the STUT varied depending on the amplitude of the injected current but remained within the gamma frequency range (36.52 ± 2.48 Hz at 100 pA and 49.03 ± 2.42 Hz at 280 pA DCI, n = 11). Importantly, the APs, arising in response to the suprathreshold pulses, were also generated in the gamma frequency range (33.59 ± 2.36 Hz at 100 pA and 50.89 ± 2.75 Hz at 280 pA, Figure 5C). Notably, the firing frequency of the STUT was significantly different to those of the FAST at both DCI levels (46.52 ± 4.43 Hz at 100 pA, *p* = 0.01 and 97.11 ± 12.30 at 280 pA DCI, *p* = 0.0008, n = 9). No significant difference was found between the subthreshold oscillations and the AP discharge frequency in the STUT during the bursts at the two DCI levels tested (*p* = 0.19 for 100 pA and *p* = 0.12 for 280 pA DCI). A comparison of the first and subsequent APs demonstrated that latter arise at more depolarized levels and become slower in their time course (Figure 5D). These results suggest that in the active network, with sufficient excitatory drive, STUT are able to generate brief epochs of gamma frequency-patterned output in the neuronal network and therefore may play an important role in the generation of gamma frequency network oscillations in the mEC.

Next, we examined the subthreshold membrane potential oscillations of STUT in the presence of the GABA_B_ receptor agonist baclofen because baclofen has a well-known effect on the attenuation of network gamma oscillations [44,45]. The baclofen completely suppressed the subthreshold oscillations in all the examined STUT (n = 4) at all the depolarization levels (Figure 5E). Moreover, this drug also strongly reduced the spiking of the STUT in the DCI range used (see also [46]). These effects were not the direct consequence of membrane potential hyperpolarization since these changes remained after biasing the membrane potential to its pre-baclofen value (Figure 5E).

### 2.6. Opioid Receptor Activation Promotes Gamma Frequency Oscillations in the mEC

**Figure 6:**
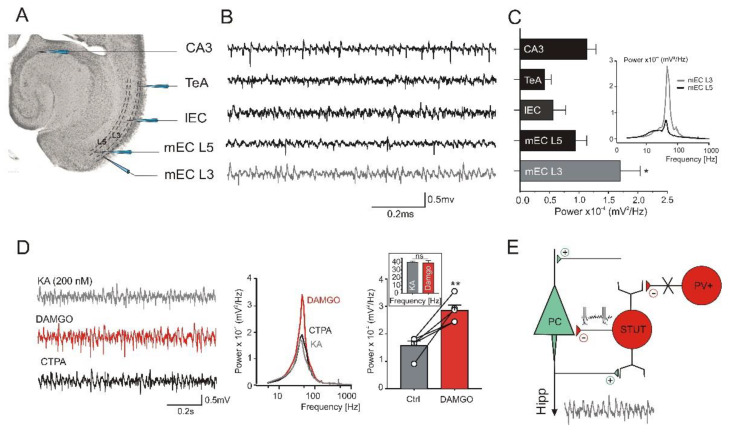
Opioid receptor agonist promotes gamma frequency oscillations in superficial mEC. (**A**,**B**) Gamma frequency oscillations in EC–hippocampal combined slices induced by low molarity of kainate. (**C**) The oscillatory activity was observed in all recorded areas but with different power. Peak power of gamma frequency oscillations within the mEC was significantly stronger in superficial layers (L3) compared to deep layer (L5). *p* value is given as * (*p* ≤ 0.05). (**D**) Effect of opioid receptor agonist damgo on network oscillations in mEC L3 (**left**). The power of gamma frequency oscillations was significantly increased in the presence of damgo, whereas the corresponding frequency remained unchanged (**right**). *p* values are given as ns (*p* > 0.05) and ** (*p* ≤ 0.01). The effect of damgo was reversed by the μ-opioid receptor antagonist, CTPA (**middle**). (**E**) Scheme with a possible explanation of μ-opioid receptor activation on the properties of STUT and network oscillations. During field oscillation the active PCs of L3 provide excitatory tonic input to STUT which cause the feedback inhibition. μ-opioid receptor activation at PV-positive IN-STUT (in red) synapses causes disinhibition of STUT, which increases their gamma frequency-patterned output to the PCs, leading to increases in the field gamma frequency oscillations.

The disinhibition of STUT by opioid receptor activation (see above) might influence the network oscillatory activity within the mEC; that is, it might enhance or promote the gamma frequency network oscillations. To test this assumption in a last set of experiments, we studied the network oscillations induced by a low molarity of kainate (KA) and tested the effect of μ-opioids on this oscillatory network activity. We first analyzed the KA-induced oscillations within the superficial (L3) and deep (L5) layers of the mEC as well as in the different areas of the combined hippocampal–EC slices, for comparison. Bath application of the low-molarity KA (200 nM) was able to generate network oscillations in the gamma frequency range in all the recorded areas. However, the power of the oscillations obtained from the different areas outside of the mEC within the same combined EC–hippocampal slices varied significantly, being most powerful in the CA3 area (1.13 ± 0.15 × 10^−4^ mV^2^/Hz), followed by the lateral EC (0.56 ± 0.27 × 10^−4^ mV^2^/Hz) and temporal association cortex (TeA) (0.42 ± 0.16 × 10^−4^ mV^2^/Hz, n = 6) (Figure 6A–C). Within the mEC, the power of the gamma frequency oscillations was significantly higher in the superficial compared to the deep layers (L3: 1.7 ± 0.34 × 10^−4^ mV^2^/Hz, n = 9 and L5: 0.9 ± 0.19 × 10^−4^ mV^2^/Hz, n = 11, *p* = 0.038; Figure 6C), depicting the mEC L3 as a strong source of network gamma.

We next studied the effects of damgo on the gamma frequency oscillation in the mEC L3 (Figure 6D). In the presence of damgo, the power of the gamma frequency oscillation increased significantly (from 1.57 ± 0.17 × 10^−4^ mV^2^/Hz to 2.85 ± 0.20 × 10^−4^ mV^2^/Hz, *p* = 0.0013, n = 5), while the corresponding frequency remained unchanged (before: 39.73 ± 1.28 Hz, after: 39.24 ± 2.65 Hz, *p* = 0.86, n = 5, Figure 6D). This effect on the oscillatory power was reversed by the μ-opioid receptor antagonist CTPA (1.7 ± 0.06 × 10^−4^ mV^2^/Hz, *p* = 0.28, n = 5, Figure 6D), again without affecting the oscillatory frequency (39.94 ± 2.78 Hz, *p* = 0.95, n = 5). These results collectively suggest that the μ-opioid system and STUT may effectively promote mEC oscillatory network activity in the gamma frequency range.

## 3. Discussion

In this study, we describe for the first time a subset of PV+ INs within the superficial mEC, STUT, with the ability to generate subthreshold membrane potential oscillations and AP firing in the gamma frequency range and the capability to support the corresponding network oscillations. Their extensive axonal arborization pattern within L2/3, where the somas and apical dendrites of the PCs are located, are important morphological prerequisites to entrain network gamma rhythm in the PC population. We suggest that, in the active network, STUT provide a gamma frequency-patterned output within the superficial layers of the mEC, promoting this network activity. μ-opioids increase the network gamma rhythms and affect the GABAergic synaptic transmission within the mEC microcircuits in a cell-specific manner. In particular, μ-opioid receptor activation leads to disinhibition of STUT and allows them to implement their gamma-patterned output, which may play a crucial role for the network oscillatory activity. Thus, we offer new insights into the neural circuit mechanisms and for their anatomical and physiological separation within the superficial mEC, which is relevant for the generation of fast network oscillations in this structure.

Within the superficial EC, L3 PCs, as the main output of this layer, form the monosynaptic temporoamonic projection to the CA1 and subiculum. These cells have functional implications for the EC–hippocampal interaction as a principal cell type relevant for gamma oscillations [14]. The examination of the morphological and electrophysiological properties of these reelin-negative L3 PCs in our study revealed specific features that are in line with our earlier work and other studies [2,3,5]. The inhibitory transmissions (IPSCs) onto these cells are only minimally sensitive to the μ-opioid receptor agonist damgo (Figure 4), in contrast to the SCs [31]. However, the μ-opioid receptor activation enhanced the gamma frequency oscillations at the network level (Figure 6), pointing towards the INs as being the source of this enhancing effect.

Immunohistochemically distinct and structurally and functionally separated, inhibitory INs were described for the mEC [17,18,19,20,21,22,23]. Among these INs, PV+ INs have been best characterized [16,22,47,48,49,50]. These INs are typically fast-spiking, non-adapting (basket or chandelier) GABAergic cells. In L2, the SCs receive strong perisomatic inhibition from fast-spiking PV+ INs [31,40,51,52] and dendritic inhibition from somatostatin-positive INs [53]. Considering that grid cells are the principal cells of the superficial mEC [53,54,55], the inhibitory networks may contribute to grid formation in this structure. This is particularly the case for PV+ INs that directly inhibit the firing of the grid cells, as well as the border and head direction cells [17,20]. However, in our study the firing patterns of the PV+ INs in the mEC are not homogenous but include two separate firing modes: high-frequency tonic firing and rhythmic or irregular bursting, stuttering [41]. Here, we show that these different firing modes belong to two morphologically distinct subtypes of PV+ INs, FAST and STUT. The axons of PV+ FAST formed the typical longitudinal branching mainly restricted to L2 and localized around the principal cell bodies of this layer (Figure 1 and Figure 2). In this study, we describe a distinct PV+ IN subtype in the superficial mEC, STUT, which comprise a new subtype of PV+ INs with their clearly distinct morphological features, which have so far been undescribed in the mEC. The axonal arborization pattern of these cells is well aligned to the perisomatic region of EC principal cells but, in contrast to FAST, targets not only L2, where the SCs are localized, but also L3, with a predominant PC population. STUT possess further unique electrophysiological properties with subthreshold membrane potential oscillations and firing in the gamma frequency range and are therefore in a position to support the generation of gamma-frequency network oscillations.

Network oscillations at the gamma frequency range (30–90 Hz) have been reported in the ECs of humans [56] and rodents, both in vivo [30,57,58] and in vitro [14,31]. Inhibition mediated by fast-spiking PV+ INs play a critical role in the generation of this oscillatory rhythm [59,60,61,62,63,64]. Two main mechanisms, in different brain areas and during different brain states, have been suggested [65,66,67]. The oscillatory activity can be mediated either by coupled excitatory and inhibitory neurons [68], i.e., the pyramidal-interneuron gamma (PING) mechanism [69], or by tonic excitation of mutually coupled inhibitory neurons, i.e., the interneuron gamma (ING) mechanism [70,71]. In the PING model, excitation of the PCs causes the innervated local INs to fire, which then, as a feedback, inhibits PCs until the inhibition fades and the next cycle can occur [67,70,72]. Gamma frequency oscillation induced in vitro by the activation of acetylcholine or KA receptors [14,67] has been suggested to be generated by a PING mechanism [67,70,72]. In accordance with such a PING mechanism, we propose that the increase in the PC firing rate in the active network [14] provides the tonic excitation of STUT and causes them to fire at their preferred gamma frequency, resulting in the initiation of rhythmic synchronous inhibition within the network and thus the generation of field gamma oscillations. Importantly, in the superficial mEC, fast-spiking INs receive excitatory synaptic potentials (EPSPs) during field gamma oscillations with lower frequency (~29 Hz) than the field gamma itself [14]. Therefore, in order to generate an output from the INs corresponding to the field gamma frequency, an additional mechanism apart from the phasic population EPSPs is required. STUT´s ability to produce subthreshold membrane oscillations and gamma frequency patterned discharge perfectly meets this requirement.

The opioid system influences network activity as well as learning and memory processes [73,74]. The PV-immunoreactive INs, which provide the majority of the local inhibition in the superficial layers of the mEC [31], express μ-opioid receptors [32,33,34]. We previously reported that the slow inhibitory synaptic potentials (hyperpolarization that lasted for up to 20 s) of the mEC L3 principal cells were sensitive to the μ-opioid receptor antagonist (naloxone), suggesting a role for opioids in its generation [3]. In the present study, analyzing the impact of the selective μ-opioid receptor agonist damgo on the (fast) inhibitory transmission onto L3 PCs, we found negligible direct effects (Figure 4). In contrast, damgo strongly reduced inhibition in the PV-positive (STUT) but not the PV-negative (REG) INs and therefore could modulate/control the excitability of the PCs indirectly via disinhibition in a cell-specific manner. This finding is in good agreement with previous observations, showing that μ-opioids modulate the excitability of PCs via an indirect process of disinhibition [75,76,77]. Hence, the damgo-induced enhancement of the gamma frequency oscillation could be explained, at least partially, by the attenuation of the inhibitory synaptic inputs onto the STUT. In this scenario, the amount of synchronous, phasic GABA release at the gamma frequency range from the STUT axon terminals to the principal cells is increased to support the generation of field oscillations. In addition, the PCs of the superficial mEC increased their firing rate during gamma oscillations [14]. Feedback activation would produce strong excitatory drive onto STUT and thus further contribute to their increased activity and oscillatory power. Hence, the two cellular mechanisms might contribute to an increase in gamma power following activation of the μ-opioid receptors: (a) disinhibition of STUT (Figure 6E) and, as a result, a stronger STUT-PC interaction; (b) feedback excitation of STUT by joint gamma-generating PCs in the active network [14].

The INs in the cerebral cortex, hippocampus, amygdala, and striatum have been suggested to possess robust frequency selectivity [41,78,79,80,81,82,83,84]. These INs show oscillations during intracellular current injection or pharmacologically induced depolarization with frequencies in the theta (4–7 Hz) or gamma (30–90 Hz) range [78,85,86,87,88,89,90]. In addition, gamma resonance in fast-spiking INs with subthreshold oscillations at membrane potentials near the AP threshold [41,78] and spike resonance [83] were reported. Importantly, IN subthreshold oscillations and resonance behavior may enhance network oscillations [91]. The membrane resonance is further thought to be important for the generation of a robust gamma rhythm that can reproduce the characteristics of both PING and ING [92]. In our earlier studies, we demonstrated the distinct firing properties of certain hippocampal inhibitory INs, along with clearly different impacts on the synchronized network activity, which appeared to correspond mainly to their intrinsic membrane properties [61,93,94]. The range of output profiles was further accompanied by distinct axonal arborization patterns and terminal field profiles [93,94].

In the mEC, voltage-dependent subthreshold membrane potential oscillations and resonance behavior in the theta frequency range were observed in the principal cells of both the superficial (L2 SCs) and the deep (L5 PCs) layers [1,26,27,95,96,97,98], suggesting a contribution to field theta rhythm generation. To the best of our knowledge, the intrinsic membrane oscillations and firing at gamma frequency range in the INs, as we reported here, have not been previously examined in the EC. Given such intrinsic membrane and firing properties, STUT are ideally suited to contribute to the rhythm generation and exact timing of the gamma oscillations in the mEC. This is a very different role than those of the REG, whose main task seems to be to provide the principal cells with tonic inhibition. REG is sensitive to the cannabinoid receptor agonist and possesses a regular firing pattern, features that are specific for regular-spiking INs such as CCK-positive cells [39] with their known ability to provide tonic rather than phasic inhibition. In contrast to STUT, the monosynaptic IPSCs in REG are only slightly sensitive to damgo, suggesting that this cell population does not receive a strong inhibitory input from PV+ INs (Figure 3).

Subthreshold membrane potential oscillations in neurons are based on different ionic currents [99,100,101,102]. In our study, we analyzed the effects of metabotropic GABA_B_ receptor activation on the intrinsic membrane and firing properties of STUT. These receptors are known to provide pre- and postsynaptic inhibitory effects in different brain areas [103]. The postsynaptic effect is typically mediated by the G protein-mediated pathway through the activation of the GIRK and TREK-2 potassium channels that result in outward potassium currents and the hyperpolarization of the cells [104]. ECs express a high density of GABA_B_ receptors [105,106,107]. STUT are able to generate voltage-sensitive subthreshold membrane potential oscillations at a moderate, physiologically relevant depolarizing level and are interrupted by clusters of AP trains at the same gamma frequencies. This interplay of subthreshold oscillations with clusters of AP trains indicates the involvement of several ionic currents. The application of baclofen strongly suppressed the intrinsic membrane oscillations and AP firing in STUT and thus significantly inhibited the neuronal excitability and output from these INs. Although the exact underlying mechanism of this effect is unclear, it is not a direct consequence of the hyperpolarizing effect of baclofen. Rather, baclofen acting via potassium channels, with its ‘shunting’ effect disrupting the oscillatory interplay of different ionic currents, is a possible mechanism, but the exact role of the specific ionic currents for the intrinsic subthreshold oscillations of STUT should be examined in future studies. Our results indicate that in the EC the GABA_B_ receptors may exert a tight control over the network oscillation at gamma frequencies, over a broad range of mechanisms, including the modulation of the neuronal excitability and the discharge pattern of STUT. Indeed, the suppression of gamma frequency oscillations has been reported in the presence of baclofen [44,45].

## 4. Materials and Methods

All animal procedures were approved by the Regional Berlin Animal Ethics Committee and were in full compliance with national regulations. Experiments were performed on P18-P25 C57Bl/6 wildtype mice and transgenic mice that expressed enhanced fluorescent protein under the control of the parvalbumin promoter [108].

### 4.1. Slice Preparation

Transverse EC–hippocampal combined slices were cut at either 300 μm (whole-cell recordings) or 400 μm (field recordings) thickness and incubated for at least 1 h in a holding ‘submerged’ or interface chamber, respectively. The slices were continuously oxygenized with carbogen and perfused with ACSF at ∼2 mL/min, at 33 ± 1 °C. For patch-clamp recordings, the slices were transferred to the recording ‘submerged’ chamber (perfused at a rate of 6–8 mL/min), at 33 ± 1°C. The solution used during cutting, incubation, and recording contained (in mM): NaCl, 129; KCl, 3; NaH_2_PO_4_, 1.25; CaCl_2_, 1.6; MgSO_4_, 1.8; NaHCO_3_, 21; glucose, 10; saturated with 95% O_2_ and 5% CO_2_, pH 7.4; and 290–310 mOsm.

### 4.2. Extracellular Field Recording

Local field potential (LFP) recordings were obtained in an interface chamber from the stratum pyramidale of the hippocampus (area CA3), the EC (mEC and lEC) and the temporal-associated cortex. Kainic acid (KA) (200 nM) was applied in the bath to induce network gamma frequency oscillations. Field oscillations were low-pass filtered at 1 kHz, digitized at 10 kHz (Digidata 1322, Axon Instruments), and analyzed with the pClamp software package (Axon Instruments). Oscillatory peak frequency was determined by averaging several consecutive Fourier transforms contained within a 20 to 30 s epoch. A Student´s *t*-test was used for statistical comparisons; differences were considered significant if *p* < 0.05. Average values are expressed as mean ± SEM.

### 4.3. Whole-Cell Recording

The patch-clamp recordings were obtained from the principal cells and INs of superficial mECs visualized by infrared differential interference contrast video microscopy. To determine the affiliation of the recorded INs, a transgenic mouse (PV GFP) was used. IN types were visualized and characterized by the presence or absence of parvalbumin. The intrinsic and firing properties of cells were measured in whole-cell current-clamp mode, as described previously [61,109]. Subthreshold membrane potential oscillations and the firing of cells were analyzed using depolarizing current injections (DCI) with different intensity (up to 280 pA). DCI intensity was chosen to depolarize the cells to a physiological range observed in the active network during a pharmacologically induced gamma frequency oscillation in vitro [61]. The voltage responses of the cells upon the hyperpolarizing and depolarizing current injection and the corresponding current–voltage (I-V) relations for each voltage response were studied.

Isolated GABAergic synaptic transmission was investigated in the presence of glutamate receptor antagonists (CNQX, 10 μM; APV, 60 μM) using electrical stimulation at the border between L2 and L3, in the close vicinity of recorded cells. Monosynaptic stimulation-evoked inhibitory postsynaptic currents (IPSCs) were recorded from cells held in a voltage clamp at −70 mV. Monosynaptic stimulation was used to analyze the IPSC amplitudes. The peak amplitudes of the IPSCs were recorded before and after application of μ-opioid and cannabinoid receptor agonists (damgo, 200 nM and WIN 55.212, 1 μM respectively). The effects of damgo and WIN were antagonized with the μ-opioid and cannabinoid receptor antagonists STAP (500 nM) and AM 251 (5 μM), respectively. μ-opioid receptor agonists and antagonists as well as GABA_A_ receptor antagonist gabazine (10 μM) were applied by bath.

Whole-cell recording pipettes (3–5 MΩ) were filled with a solution containing (in mM): K-gluconate, 70; KCl, 70; NaCl, 2; ATP-Mg, 4; GTP-Na, 0.3; EGTA, 4; HEPES, 10; and plus biocytin, 0.5% (pH 7.4 and 290 mOsm). A Multiclamp 700B amplifier and pClamp software (Axon Instruments) were used for current- and voltage-clamp recordings. The seal resistance before establishing the whole-cell mode was ≥2 GΩ. The series resistance (range 12–18 MΩ) was not compensated but was repeatedly monitored during the experiment by measuring the amplitude of the capacitive current in response to a −10 mV pulse. Experiments in which the series resistance increased by >20% were discarded. Signals were low-pass filtered at 5 kHz, digitized at 10 kHz (Digidata 1322), and analyzed using pClamp software. Electrophysiological identification was confirmed post hoc by immuno- and biocytin staining.

### 4.4. Immunolabeling

The immunolabeling for recorded cells was similar to that described in [109]. IN and the principal cell types in the EC were visualized and identified using antibodies against parvalbumin and reelin, respectively. For immunolabeling of the INs, the slices were immersed overnight in a fixative solution containing 4% paraformaldehyde in 0.1 M phosphate buffer (PB), washed three times in 0.1 M PB, and subsequently in 0.1 phosphate-buffered saline (PBS; pH 7.3). The slices were then incubated in PBS containing 0.3% Triton X-100, 10% goat serum, and mouse-on-mouse blocking reagent for 1 h at room temperature. To visualize the PV- and reelin-containing cells, we used antibodies against PV (rabbit, Swant, PV27) and reelin (mouse, Millipore, AB5364 clone G10) diluted 1:1000 and 1:2000, respectively, in PBS containing 5% goat serum and 0.3% Triton X-100. The slices were incubated with primary antibodies for 48 h at room temperature. After rinsing three times in PBS, the sections were incubated in the PBS solution containing 0.3% Triton X-100, 5% goat serum, goat anti-rabbit conjugated with Alexa fluor 488 (for PV, Invitrogen Corporation, Carlsbad, CA, USA) and Alexa fluor 594 (for reelin, Invitrogen Corporation, Carlsbad, CA, USA), diluted 1:500. To visualize the biocytin-filled cells by fluorescence microscopy, avidin conjugated Alexa fluor 350 diluted 1:500 (Invitrogen Corporation, Carlsbad, CA, USA) was added. The slices were mounted on glass slides in the glycerol-based, aqueous mounting Vectashield (Vector Laboratories) under coverslips at 24 h after incubation with the secondary antibodies. The labelled cells were visualized using 20× and/or 60× objectives. 

### 4.5. Nissl Staining

Nissl staining was used to highlight the structural features and distribution of neurons in the superficial mEC. The sections were mounted on gelatin-coated slides and allowed to air-dry overnight. The mounted sections were rehydrated in distilled water and submerged in 0.2% cresyl violet solution. The sections were rinsed in distillate water and dehydrated in a graded series of ethanol, immersed in xylene, mounted in DPX, and coverslipped. 

### 4.6. Biocytin Staining

To verify the identity of the recorded neurons, they were filled with biocytin and fixed in 4% paraformaldehyde. The slices were processed as described previously [109,110]. The slices were washed three times in 0.1 M PB. The avidin–biocytin complex reaction (Vectastain ABC kit, BIOZOL Diagnostica, Eching, Germany) took place overnight at 4 °C in the presence of 0.3% Triton X-100 (Sigma-Aldrich, Taufkirchen, Germany). Afterwards, the sections were rinsed several times before development with 0.02% diaminobenzidine in 0.1 M PB. The reaction product was intensified with 0.5% OsO_4_, and the sections were mounted and coverslipped. The neurons were reconstructed using a Neurolucida 3D reconstruction system (MBF Bioscience, Williston, ND, USA).

In Sholl analysis, concentric nested spheres, each 100 μm in diameter, were centered around the soma of each reconstructed cell (first shell: 0–100 μm, second shell: 100–200 μm, etc.). Axonal branching was then quantified by Neurolucida using the total axonal length in a given shell, while the axonal parts in the smaller shells were ignored. Finally, for each cell the summed length values per shell were normalized to the maximum summed length value of all the shells (set equal to 100) and averaged across all the cells of FAST, STUT, and REG, respectively.

## Data Availability

The data presented in this study are available in the article.
